# Polymer Electrolyte Membranes Prepared by Graft Copolymerization of 2-Acrylamido-2-Methylpropane Sulfonic Acid and Acrylic Acid on PVDF and ETFE Activated by Electron Beam Treatment

**DOI:** 10.3390/polym11071175

**Published:** 2019-07-11

**Authors:** Xi Ke, Yufei Zhang, Uwe Gohs, Marco Drache, Sabine Beuermann

**Affiliations:** 1 Institute of Technical Chemistry, Clausthal University of Technology, Arnold-Sommerfeld-Strasse 4, 38678 Clausthal-Zellerfeld, Germany; 2 Institut fur Leichtbau und Kunststofftechnik, Technische Universitat Dresden, Hohlbeinstrabe 3, 01307 Dresden, Germany

**Keywords:** polymer electrolyte membranes, ETFE, PVDF, acrylic acid, 2-acrylamido-2-methylpropane sulfonic acid, fuel cells

## Abstract

Polymer electrolyte membranes (PEM) for potential applications in fuel cells or vanadium redox flow batteries were synthesized and characterized. ETFE (poly (ethylene-alt-tetrafluoroethylene)) and PVDF (poly (vinylidene fluoride)) serving as base materials were activated by electron beam treatment with doses ranging from 50 to 200 kGy and subsequently grafted via radical copolymerization with the functional monomers 2-acrylamido-2-methylpropane sulfonic acid and acrylic acid in aqueous phase. Since protogenic groups are already contained in the monomers, a subsequent sulfonation step is omitted. The mechanical properties were studied via tensile strength measurements. The electrochemical performance of the PEMs was evaluated by electrochemical impedance spectroscopy and fuel cell tests. The proton conductivities and ion exchange capacities are competitive with Nafion 117, the standard material used today.

## 1. Introduction

A polymer electrolyte membrane fuel cell (PEMFC) may be seen as an energy converter that combines the characteristics of low pollution, low noise level, renewable energy and high efficiency, etc. [1,2,3,4,5,6]. A key part of the PEMFC is the membrane electrolyte assembly (MEA) [7,8,9,10], which consists of a polymer electrolyte membrane (PEM), catalyst, and two electrodes as main components. The PEM is a selective permeable membrane that separates the gases serving in both half cells while allowing for transfer of protons, which is associated with a variety of requirements. First of all, the PEM must exhibit high proton conductivity and low electrical conductivity and at the same time low gas permeability. In addition, mechanical and thermal stability is needed. Furthermore, low cost is very important for acceptance of PEMFCs and a wide use. Depending on the operating temperature the membranes for PEMFC are divided into high-temperature polymer electrolyte membranes (HT-PEM) and low-temperature polymer electrolyte membranes (LT-PEM). With phosphoric acid frequently being used as proton conductor, HT-PEMs show a relatively high operating temperature in a range from 160 to 200 °C [11]. Aiming for better water retention other approaches have also been explored [12]. LT-PEM are mostly based on a stable fluorinated or polyaromatic backbone polymer, e.g., poly(ethylene-alt-tetrafluoroethylene) (ETFE) [13,14,15,16], poly (vinylidene fluoride) (PVDF) [17,18,19] or poly(ether ether ketone) (PEEK) [20,21,22,23]. Protogenic functional groups such as sulfonic acid groups are attached to these matrices [24,25,26,27,28,29]. Water is used as a protic solvent. At an operating temperature above the boiling point of water, the PEM is dehydrated, which increases the internal resistance. Therefore, the operating temperature of the LT-PEM is normally not higher than 100 °C [30,31,32,33]. The Nafion^®^ membrane is the most commonly used LT-PEM in fuel cells [34,35,36,37,38]. Previously, we reported a different type of polymer electrolyte membranes, which was prepared by activation of ETFE base material with electron beam (EB) treatment, subsequent graft radical copolymerization of methacrylate monomers on ETFE followed by sulfonation to introduce protogenic groups [39,40]. The electrochemical properties of these materials are well suited for applications in LT-PEMFC or vanadium redox flow batteries.

Rather than introducing protogenic groups into the material in an additional process step that follows the polymerization it is highly advantageous to use monomers that already carry protogenic groups, e.g., such as 2-acrylamido-2-methylpropane sulfonic acid (AMPS). Previously, it was reported that the use of AMPS requires the graft copolymerization with another monomer to achieve sufficient mechanical stability and to limit swelling of the material [41]. More recently, AMPS was used to prepare PEMs based on sulfonated poly (arylene ether ketone sulfones) [42]. 

In this work, a synthetic strategy for the preparation of LT-PEM is presented, which does not require an extra sulfonation step. Activated PVDF or ETFE base material is grafted with two monomers already carrying protogenic groups, AMPS and acrylic acid (AA) via radical copolymerization. Moreover, these monomers allow for graft copolymerization in water, thus, avoiding the use of organic solvents.

## 2. Materials and Methods

### 2.1. Materials

Poly (ethylene-alt-tetrafluoroethylene) (ETFE, Nowofol, Nowoflon ET-6235Z, Siegsdorf, Germany) and poly (vinylidene fluoride) (PVDF, Nowofol, PVDF homopolymer, Siegsdorf, Germany) films with a thickness of 50 µm were used as a substrate. Acrylic acid (AA, 99%, Aldrich, Zwijndrecht, The Netherlands) was purified using an inhibitor remover column (Aldrich, St. Louis, USA) and stored at 4 °C in the dark before use. 2-Acrylamido-2-methylpropane sulfonic acid (AMPS, 99%, Aldrich, St. Louis, MO, USA) and sulfuric acid (98%, Merck, Darmstadt, Germany) were used without further purification. Deionized water obtained from the GENO^®^ OSMO. MSR-tronic Typ 100 (Gruenbeck Wasseraufbereitung, Donau, Germany) is used as solvent for the graft copolymerization. Tashiro is a mixed indicator of 0.1 wt. % methylene blue (Merck, Darmstadt, Germany ) and 0.03 wt. % methyl red (Honeywell, Seelze, Germany) dissolved in ethanol (97%, Nordbrand Nordhausen, Nordhausen, Germany) [43]. 

### 2.2. Preparation of the PEM

Firstly, the ETFE or PVDF backbone material was activated by electron beam treatment with 50 to 200 kGy and stored at −30 °C prior to grafting. The details are given elsewhere [44]. The graft polymerizations were carried out in a double-walled glass reactor (100 mL volume) equipped with a condenser under continuous nitrogen flow for removal of oxygen and mixing of the reaction mixture. The preparation of sample AAE500 serves as an example for a typical synthesis: Firstly, the monomers (16 g AMPS and 5.6 g AA) and the solvent H_2_O (80 g) were added to the reactor. After flushing with nitrogen for 10 min, the reaction solution was heated to the reaction temperature of 80 °C. Then, the activated ETFE or PVDF with a precisely known weight was introduced into the reactor and the reaction proceeds for 3.5 h. To determine the degree of grafting the grafted films were dried in vacuum at 60 °C until constant weight was reached. *DG* was calculated according to Equation (1):

(1) $$DG\;=\;\frac{m_{p}\;-{\;m}_{\mathrm{ETFE}}}{m_{\mathrm{ETFE}}}$$

*m*_ETFE_ and *m*_p_ are the masses of the film before and after the grafting, respectively. For protonation, the membrane was washed with deionized water and soaked in 0.5 M H_2_SO_4_ for 24 h.

### 2.3. Fuel Cell Tests

The fuel cell tests were carried out at 40 and 50 °C with a stoichiometric gas excess ratio of 1.1 for both gases (λ_H2_, anode = 1.1, λ_O2_, cathode = 1.1) in a single fuel cell device as detailed previously [44].

### 2.4. Characterization

#### 2.4.1. Elemental Analysis

To determine the composition of the graft polymer, the content of carbon, hydrogen, nitrogen and sulfur was determined using a vario EL instrument from Elementar Analysesysteme GmbH, Langenselbold, Germany. The calibration was carried out with sulphanilic acid. The fluorine-containing samples are measured in C, H, N, S mode at a furnace temperature of 1150 °C.

#### 2.4.2. Mechanical Analysis

Mechanical properties of the PEMs were determined using a Zwick Z2.5/TN1S (Zwick Roell AG, Ulm, Germany) instrument with the analysis software testXpert 6.0 (Zwick Roell AG, Ulm, Germany). The polymer membranes are soaked with deionized water at room temperature for 24 h. Then, they were cut off in stripes of 1 cm × 10 cm. The measurements were performed at 20 °C at a crosshead speed of 10 mm·min^−1^. Ultimate tensile strength (*UTS*), elastic modulus (*E*) and elongation at break (*ε*) were obtained.

#### 2.4.3. Ion Exchange Capacity

To determine the number of acid groups in the polymer membrane, the PEMs were analyzed further. A piece of the membrane (3 cm × 3 cm) was immersed in 100 mL 0.01 M NaOH solution overnight. Thereafter, 10 mL of this solution was titrated with 0.005 M H_2_SO_4_ against Taschiro indicator. The color changes from gray (pH = 5.2) to purple (pH < 5.2) [45]. Since NaOH can react with CO_2_ in the air, analysis of a blank sample (0.01 M NaOH without the membrane) is required. After the titration, the ion exchange capacity (*IEC*) of the membrane is calculated according to Equation (2) [39].

(2) $$\mathrm{IEC}\;[\mathrm{mol}\cdot g^{-1}]\;=\;\frac{V_{b}-V}{m_{\mathrm{dry}}}\;\cdot c_{\mathrm{NaOH}}$$

where *m*_dry_ is the mass of the dry PEM, *c*_NaOH_ the concentration of the NaOH solution, *V*_b_ and *V* are the consumed volume of the blank solution and the solution with the PEM, respectively. 

#### 2.4.4. Electrochemical Impedance Spectroscopy

Further, the PEM was examined with an electrochemical impedance spectroscopy (EIS) instrument IviumStat (Ivium Technologies, Eindhoven, The Netherlands) in the frequency range between 1 MHz and 10 Hz. During the measurement, 0.16 cm^2^ of the polymer membrane was located between two stainless steel electrodes, which was screwed together with a torque of 0.5 Nm.

#### 2.4.5. Differential Scanning Calorimetry (DSC) 

A device DSC 820 from METTLER TOLEDO (Gießen, Germany) was used for the DSC measurements. Before the actual measurement, a complete heating and cooling cycle of −80 to 200 °C was performed. The heating and cooling rate is 10 K∙min^−1^.

## 3. Results and Discussion

**Scheme 1:**
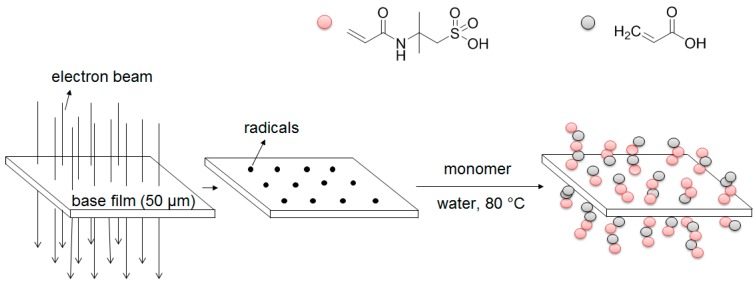
Illustration of the synthetic route for PEM preparation.

The PEMs were synthesized as illustrated in Scheme 1. Firstly, the base material, either PVDF or ETFE, was activated via EB treatment. The activated material may be stored at −30 °C up to at most 6 months [46]. In the second step the monomers acrylic acid and 2-acrylamido-2-methylpropane sulfonic acid were grafted onto the base material. The graft copolymerization of AMPS and AA is influenced by the following parameters: EB dose, type of base material, the volume fraction of monomer, *V*_m_, in the reaction mixture, and mole fraction of AMPS, *f*_AMPS_, in the monomer feed. 

### 3.1. PEM Synthesis

**Figure 1:**
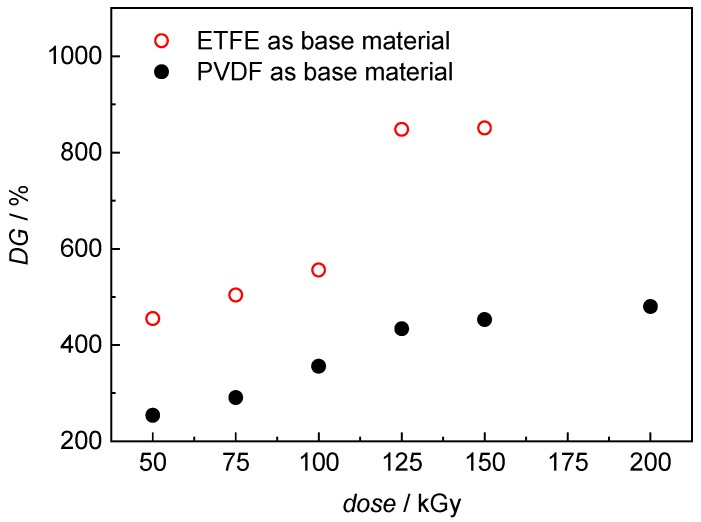
Variation of *DG* with pre-irradiation dose for graft copolymerizations on PVDF and ETFE with *f*_AMPS_ = 0.5, *V*_m_ = 0.2, reaction time 4 h at 80 °C.

Previously, graft copolymerization on ETFE after pre-activation via EB treatment was studied in detail [39,44]. It was shown that EB doses ranging from 40 to 100 kGy in the activation step are well suited to achieve high degrees of branching (*DG*) and very good proton conductivity of the PEMs. In order to obtain information on the process using PVDF, the base material was exposed to doses ranging from 50 to 200 kGy. Subsequently, all activated samples were used in graft copolymerizations with AA and AMPS at ostensibly identical conditions with a reaction time of 4 h, the AMPS molar ratio in the monomer feed is 0.5, the volume fraction of monomer *V*_m_ is 0.2, and the reaction temperature 80 °C. FTIR spectra given as Figure S1 of the Supporting Information indicate that both monomers were incorporated into the graft copolymer. As illustrated in Figure 1 *DG* increases from 250% for 50 kGy to 425% for 125 kGy activation. Further increase in dose leads to a less pronounced increase in *DG* up to 475% at 200 kGy. For comparison, copolymerizations on ETFE were carried out at identical conditions. As indicated in Figure 1, the use of ETFE as base material leads to substantially higher *DG* values. 

**Figure 2:**
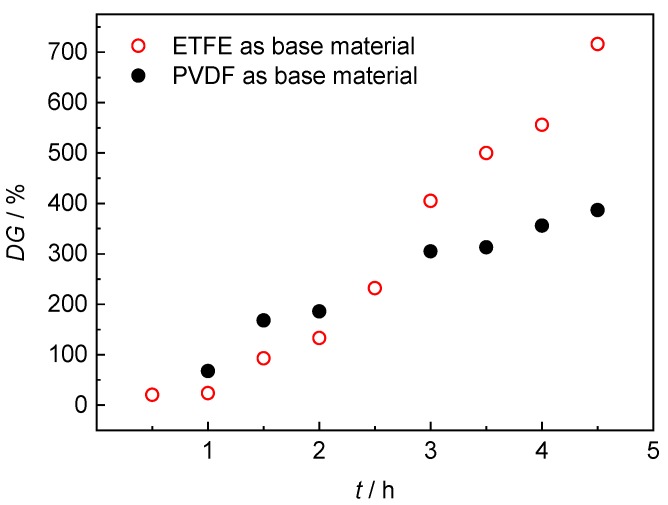
Impact of reaction time on *DG* for copolymeriaztions with *f*_AMPS_ = 0.5 and *V*_m_ = 0.2 at 80 °C. PVDF and ETFE were treated with 100 kGy.

The impact of reaction time on the degree of grafting, *DG*, of the membranes was investigated for times ranging from 0.5 to 4.5 h for ETFE and PVDF as base material activated with a dose of 100 kGy. Copolymerizations were carried out with *f*_AMPS_ = 0.5 and *V*_m_ = 0.2 at 80 °C. The results are given in Figure 2. As expected, the grafting degree increases with reaction time. 

The data in Figure 2 indicates that for both base materials similar values for *DG* were obtained up to a reaction time of 2.5 h. Then, *DG* for ETFE as base material becomes significantly higher, which is reflected by the maximum values of 716% and 400% for ETFE and PVDF as base material, respectively. The differences observed for both base materials may be seen to be due to differences in crystallinity of ETFE and PVDF. According to DSC measurements of the original ETFE material and of ETFE after applying 100 kGy the polymer is fully amorphous. On the contrary, virgin PVDF and PVDF treated with 100 kGy are associated with around 22% of crystallinity. It is suggested that the radicals in the amorphous ETFE are more easily accessible for the monomer molecules than in the partially crystalline PVDF base material. 

The rate for the copolymerization of AMPS and AA is significantly higher than for previously investigated systems where two methacrylate monomers, namely glycidyl methacrylate (GMA) and 2-hydroxyethyl methacrylate (HEMA), were grafted onto pre-activated ETFE. With GMA and HEMA as comonomers a reaction time of 3 h was required to achieve a *DG* of 170, whereas with AA and AMPS a *DG* of 205% is obtained already after two hours at comparable reaction conditions. This observation may be explained on the basis of radical polymerization kinetics. Generally, propagation rate coefficients, *k*_p_, are higher for acrylate-type monomers such as AMPS and AA compared to the methacrylate-type monomers HEMA and GMA [47,48,49]. Further, PEMs prepared with GMA and HEMA require an organic solvent for the copolymerization and a two-step sulfonation process to introduce the protogenic groups, whereas grafting of AA and AMPS avoids an additional sulfonation step. 

In another set of graft copolymerizations on PVDF the monomer composition was changed from 30 to 100 mol. % acrylic acid. *V*_m_ was 0.2, the reaction time 4 h, and the temperature 80 °C. *DG* increases from 31% to 946% upon increasing the acrylic acid content from 30 mol. % to only acrylic acid. The strong increase in *DG*, which is due to a significant increase in polymerization rate, may be explained by significantly higher *k*_p_ values for AA compared to AMPS [47,48]. On the contrary, the conductivity increases to a value of 53.4 for 60 mol. % AA (*DG* = 456%) followed by a lowering to 2.2 mS∙cm^−1^ for AA only. All data are listed in Table S1 of the Supporting Information.

Another point to consider is the amount of both monomer moieties incorporated into the PEM. Based on elemental analysis, an equimolar amount of AMPS and AA in the feed, which is used in the remainder of this publication, results in an AMPS content of 0.27 in the copolymer. This finding is in reasonable agreement with a value of 0.39 calculated with the reactivity ratios of 0.19 for AMPS and 0.86 for AA [50]. In both cases AA is preferentially built into the copolymer. The discrepancies may be due to the differences in polymerization conditions. The literature data was derived from a redox initiated radical copolymerization of AMPS and AA in water at 40 °C. In this work the monomers were grafted onto activated support materials without initiator. Thus, the accessibility of the reactive sites inside the membrane matrix is more or less limited for the monomer molecules. The smaller AA molecule may reach the trapped radicals more easily than the larger AMPS molecules. In contrast, polymerizations in solution [47] are not associated with diffusion limitations for approaching the radical chain end. In addition, AMPS monomer units carry charges leading to repulsion once a monomer approaches a polymeric radical, also explaining preferential incorporation of AA into the copolymer.

Further, the impact of the monomer volume fraction on the polymerization results with PVDF as base material was investigated. For equimolar amounts of both monomers *V*_m_ was varied from 10 to 30 vol. %. Higher monomer fractions are not feasible, since polymerization may occur in the liquid phase due to transfer of radicals to the monomers or solvent leading to polymerization in the reaction mixture [44]. *DG* increased from 28% to 1335% and the conductivity from 0.3 to 93.5 mS∙cm^−1^ in going from 10 to 30 vol. % of monomer, respectively. The details are given in Table S2 of the Supporting Information. 

At the highest *DG* the largest number of functional groups required for proton conductivity is added to the synthesized membrane, however, the mechanical stability of the PEMs decreases with *DG* duo to the reduced fraction of backbone material in the PEM. Thus, rather than using PEMs with the highest *DG,* for further mechanically and electrochemical characterization the membranes AAE500 (*DG* of 500%) and AAE205 (*DG* of 205%) based on ETFE as well as AAP305 (*DG* of 305%) and AAP421 (*DG* of 421%) synthesized on PVDF were selected. For comparison, two PEMs prepared via grafting GMA and HEMA onto ETFE with a *DG* of 170% (sample GH170) and 220% (GH220) were studied [39].

### 3.2. Mechanical Analysis

**Figure 3:**
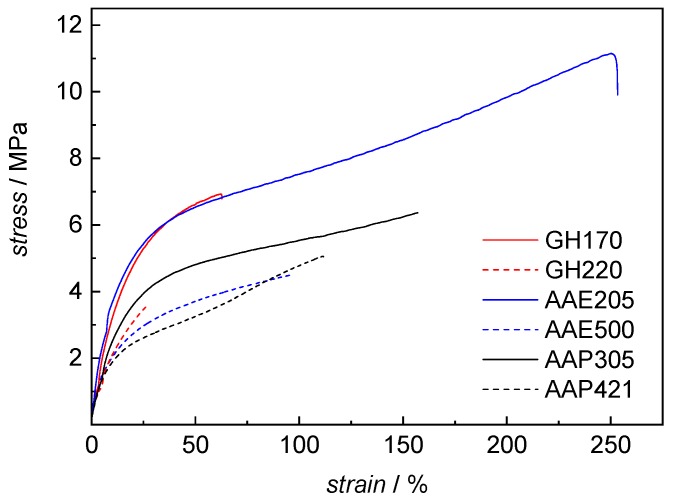
Tensile strength test of ETFE- and PVDF-based polymer membranes.

To determine the mechanical stability of the PEMs tensile strength tests with the wet samples were performed. The impact of the type of graft copolymer and the fraction of either ETFE or PVDF contained in the PEM was investigated. The results are shown in Figure 3 and summarized in Table 1. Further, Table 1 gives the water uptake, *WU*, and the ETFE or PVDF fraction of each sample. *WU* is calculated according to (*m*_wet_ − *m*_dry_)/*m*_dry_ with the masses *m*_wet_ and *m*_dry_ of the wet and dry samples, respectively. The ETFE or PVDF content was calculated from *DG* and *WU*. It is evident from the data in Table 1 that the elongation at break for samples prepared with AMPS and AA is significantly higher than for PEMs synthesized with the methacrylate monomers. Moreover, *ε* increases with the amount of residual ETFE or PVDF, the type of base material has no significant impact. 

Further, it is clearly observed that the fraction of ETFE or PVDF in the PEM strongly determines the ultimate tensile strength: both samples with an ETFE fraction of at least 10% (AAE205 and GH170) show very similar stress strain curves and are associated with the highest ultimate tensile strength (*UTS)* values of at least 6.93 MPa. The stress strain curve for AAP305 with a PVDF content of 8.9% lies below the two above-mentioned stress strain curves and an *UTS* of 6.36 MPa is calculated. The smallest *UTS* with a value close to 3.6 MPa is found for GH220. The type of base material does not have a significant impact. The E moduli show an increase with the fraction of base material for a given combination of comonomers and base material. However, the results show no common trend. On the contrary, all data determined for the water uptake follow a general trend of decreasing *WU* with increasing fraction of base material. The data for *ε UTS*, and *WU* listed in Table 1 are plotted as a function of the residual fraction of base material in Figure S2 of the Supporting Information. 

The results in Table 1 indicate that the elongation at break is significantly higher for the membranes prepared with AA and AMPS, despite the fact that in two cases the fraction of base material is lower than for GH170 and GH220. The good mechanical stability of these PEMs is suggested to be due to the fact that both monomers belong to the acrylate monomers, whereas GMA and HEMA are methacrylates. While the latter show only very little transfer to polymer, it is well known that radical polymerizations of acrylate monomers are associated with significant intra- and intermolecular chain transfer to polymer leading to branched polymer [51]. The branched material may explain the comparably high mechanical stability. This explanation is in line with a report by Faturechi et al., who observed that the presence of poly(acrylic acid) has a positive influence on the mechanical property of composite materials [52].

### 3.3. Electrochemical Properties

**Figure 4:**
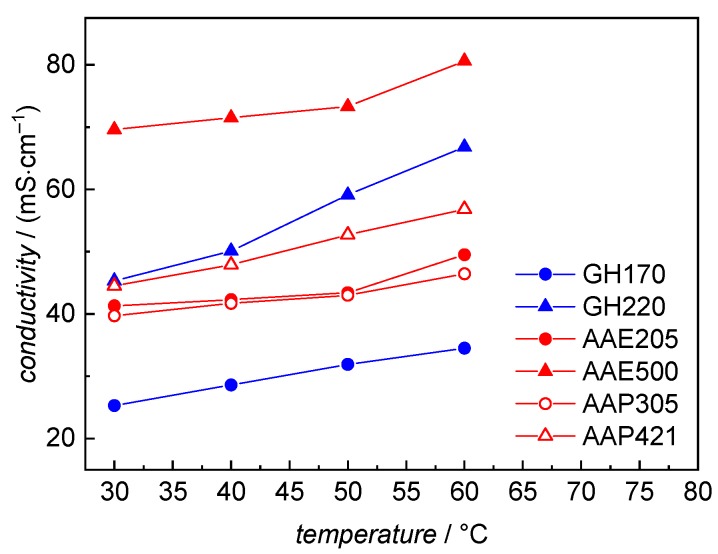
Conductivities for temperatures between 30 and 60 °C derived from impedance spectroscopy.

Electrochemical impedance spectroscopy was performed for samples GH170, GH220, AAE205, AAE500, AAP305, and AAP421 to derive the conductivities. The results are given in Figure 4. As expected, all conductivities rise with increasing temperature. GH170 shows the lowest conductivities with a maximal conductivity of 34.5 mS·cm^−1^ at 60 °C. For membrane AAE500 the highest conductivities are derived. Compared to GH170 a twice as high value of 80.6 mS·cm^−1^ is observed at 60 °C. For the other four samples rather similar conductivities, *σ* between 39.7 and 45.3 mS·cm^−1^ were determined at 30 °C. At 60 °C the values for *σ* are more different with the lowest value of 46.4 and the highest value of 66.8 mS·cm^−1^ derived for AAP305 and GH220, respectively. While *σ* values for AAE205 and AAP305 are very close for all temperatures, a significant difference in *σ* for AAE205 and GH170 with rather similar degrees of grafting is found: *σ* is significantly higher for AAE205 than for GH170: 49.6 compared to 34.4 mS·cm^−1^ at 60 °C, respectively. The corresponding value of Nafion 117 is 54.3 mS·cm^−1^ at 60 °C [39]. The conductivities for AAE205, AAE500, and GH170 are in line with the ion exchange capacities (*IEC*). For AAE500 and AAE205 excellent *IEC* values of 5.71 and 4.64 mmol·g^−1^ are determined via titration with Tashiro indicator, while a value of 2.06 mmol·g^−1^ is obtained for GH170. The differences in the *IEC* are explained by the differences in *DG* of the samples. At a higher *DG* the membrane contains a higher fraction of graft polymer with the corresponding proton-conducting groups. For PVDF-based PEM, the *IEC* was not determined, because PVDF is not stable in NaOH. 

**Figure 5:**
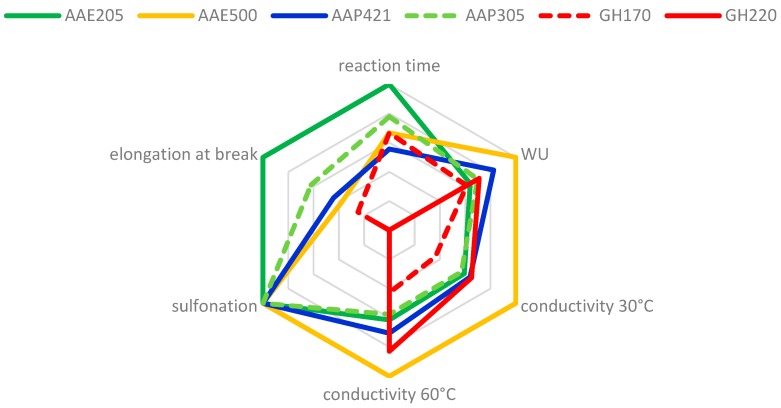
Radar diagram for important aspects related to PEM preparation and properties with the center being the least favorable and the outer circle being the most desirable condition. Further details are given in the main text.

The different contribution of the functional groups to the conductivity is important to note. Table S1 gives *DG* and conductivities for different monomer compositions. While the values of *DG* for 90 and 100 mol. % AA in the monomer feed are rather similar with 970% and 946%, respectively, the conductivities are strongly reduced from 23.3 to 2.2 mS·cm^−1^. Thus, the data suggests that the contribution of the carboxylic acid groups of the AA moieties to the conductivity is only minor. Still, the presence of AA in the monomer feed is important, since AA has a positive influence on the mechanical properties of the membranes. Another influence on conductivity is the water absorption, which increases with the degree of grafting. In order to compare the results for all PEMs listed in Table 1 important properties, such as conductivity at 30 and 60 °C, elongation at break, water uptake, and synthesis parameters, such as need for sulfonation and the reaction time are combined in one radar diagram displayed in Figure 5. The data are ratios to the maximum values for *σ*, *ε*, and *WU*. In case of the reaction time, *t*, the lowest value of 2.5 h used so far for sample AAE205 is considered as optimum. For the sulfonation process 1 is used for no sulfonation and 0 for sulfonation required, respectively. The diagram illustrates that high values are associated with limited elongation at break. It remains to be tested which values for the elongation at break are actually required for fuel cell applications. Previous experiments using PEMs prepared with other comonomer systems showed that rather low values of 20% are feasible [39]. 

Recently, PEMs similar to GH220 and GH170 were reported to be well-suited for applications in VRFBs [39]. The properties of the ETFE- and PVDF-based membranes grafted with AMPS and AA are similar to GH220 and GH170. Thus, it is anticipated that the PEM *σ* s obtained via graft copolymerization with AMPS and AA may be attractive for applications in VRFBs, too. 

### 3.4. Fuel Cell Tests

**Figure 6:**
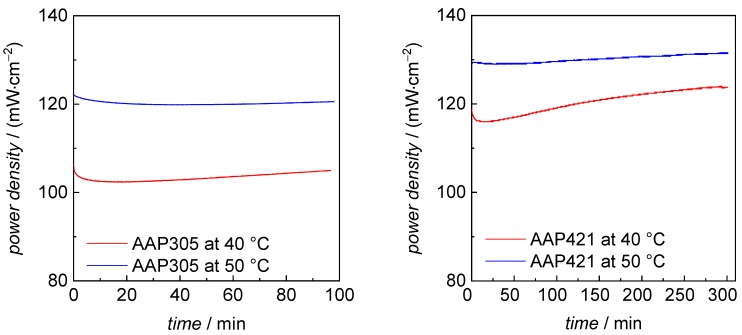
Power densities for membranes AAP305 and AAP421 determined in fuel cell tests at the indicated temperatures.

In the previous sections it was demonstrated that PEMs prepared via copolymerization of AMPS and AA on pre-activated ETFE and PVDF have promising mechanical and electrochemical properties. Moreover, visual inspection shows that the PVDF based PEMs are more flat and more smooth, which facilitates handling and contacting during assembling of the fuel cell. It remains to be tested how these membranes perform in a fuel cell. While PEMs prepared with ETFE as base material were already tested, here PEMs AAP305 and AAP421 synthesized with PVDF as material were selected for application in a low temperature fuel cell. Firstly, the fuel cell was operated with a constant current of 200 mA∙cm^−2^ for 90 min in case of AAP305 and for AAP421 for 300 min. The measurements were carried out at 40 and 50 °C. The power densities are plotted in Figure 6. In general, the power densities are rather constant with time. As expected, for both membranes the power density is higher for fuel cell operation at 50 °C. In addition, it is seen that the power density is higher for AAP421, the membrane with the higher *DG* and consequently the higher number of sulfonic acid groups incorporated. For comparison, for Nafion 117 at 50 °C a value of 122 mW∙cm^−2^ was determined in the same fuel cell at identical operating conditions after 90 min [44].

**Figure 7:**
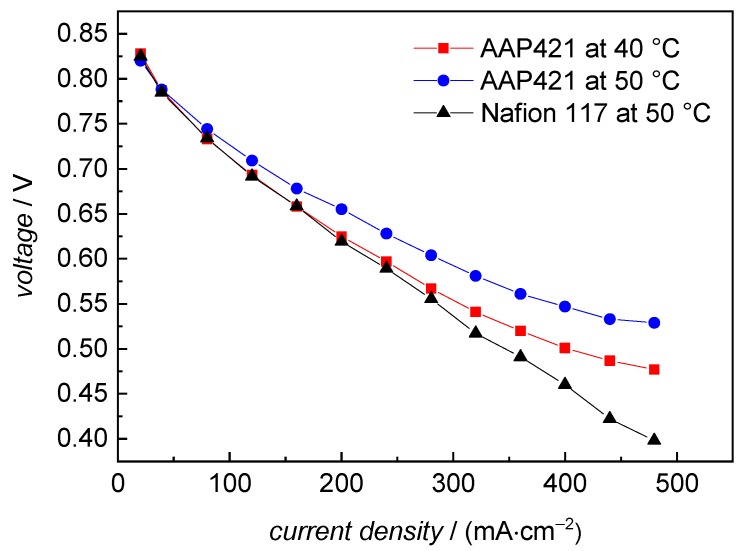
Polarization curves measured for AAP421 in a fuel cell at 40 and 50 °C. Data for Nafion 117 are taken from [44].

In addition to power densities, fuel cell tests of PEM AAP421 were carried out at 40 and 50 °C to measure polarization curves. The data is depicted in Figure 7. For comparison results obtained with Nafion 117 at 50 °C are included. The voltage—current density curves obtained for AAP421 show a slight curvature, the Nafion data show a linear dependence. The data points between 120 mA∙cm^−2^ and 320 mA∙cm^−2^ were used to calculate the Ohmic resistance. The resulting values are 26 and 23 mΩ at 40 and 50 °C, respectively. For Nafion 117 a resistance of 34 mΩ is calculated. These data indicates that the Ohmic resistances of the PVDF-based PEMs are compatible with the currently used material.

## 4. Conclusions

Using acrylic acid and 2-acrylamido-2-methylpropane sulfonic acid for graft copolymerizations on pre-activated ETFE and PVDF allows for the preparation of PEMs with significantly better mechanical stability compared to previously prepared PEMs with glycidyl and hydroxyethyl methacrylate. The improved mechanical stability is suggested to be due to chain transfer to polymer processes occurring during the copolymerization with acrylic acid as comonomer. These transfer events result in branched material. Further, the electrochemical properties are excellent with respect to a proton exchange capacity as high as 5.71 mmol·g^−1^ and a conductivity of up to 80.6 mS·cm^−1^ at 60 °C. 

The use of ETFE and PVDF as base materials shows that grafting of ETFE leads to higher degrees of branching. This finding is suggested to be due to the fact that ETFE is amorphous. Thus, the ETFE films grow better in the graft polymerization compared to the PVDF film and the ETFE based PEMs reach higher *DGs*. In general, both types of PEMs show good conductivity and sufficient mechanical stability. However, the PVDF PEMs have a flatter surface, which favors contacting of the PEM in the fuel cell. For this reason the PVDF based PEMs show better performance in the fuel cell measurement.

In addition to the excellent PEM properties, the synthetic strategy introduced has several advantages compared to previous work on the syntheses of LT-PEMs. The choice of monomers avoids the necessity of subsequent sulfonation reactions. Secondly, the use of water as solvent is preferable to organic solvents such as *N*,*N*-dimethyl formamide used before. Last but not least the acrylate-type monomers are known to polymerize very quickly in water. Together with a higher reaction temperature of 80 °C the reaction times are significantly reduced compared to previous work. 

## Figures and Tables

**Table 1:** Result of the tensile strength tests, water uptake *WU* and fraction of base material.

Sample	Base Material	*UTS* [MPa]	*E* Modulus [MPa]	*ε* [%]	*WU* [wt. %]	*Content of Base Material* [wt. %]
GH170	ETFE	6.93	43.0	62.7	155	10
GH220	ETFE	3.57	23.6	27.0	180	7.7
AAE205	ETFE	11.2	42.1	253	162	13
AAE500	ETFE	4.49	36.7	95.8	253	4.8
AAP305	PVDF	6.36	29.5	157	177	8.9
AAP421	PVDF	5.02	26.0	112	209	6.2

## Supplementary Materials

The following are available online at https://www.mdpi.com/2073-4360/11/7/1175/s1, Table S1: impact of monomer feed composition on degree of branching and conductivity; Table S2: impact of monomer mole fraction on degree of branching and conductivity; Figure S1: elongation at break, water uptake and ultimate tensile strength as a function of the fraction of base material in the PEM.
