# Supplementary material for: Polymer Electrolyte Membranes Prepared by Graft Copolymerization of 2-Acrylamido-2-Methylpropane Sulfonic Acid and Acrylic Acid on PVDF and ETFE Activated by Electron Beam Treatment
Source: Polymers (Basel). 2019 Jul 11;11(7):1175. doi: 10.3390/polym11071175 (PMC6680964; doi:10.3390/polym11071175)
Supplement: Supplementary file 1 [file polymers-11-01175-s001.pdf]

Supporting Information

Polymer electrolyte membranes prepared by aqueous graft copolymerization of 2–acrylamido–2–methylpropane sulfonic acid and acrylic acid on PVDF and ETFE activated by electron beam treatment

Xi Ke, Yufei Zhang, Uwe Gohs, Marco Drache, Sabine Beuermann

Variation of monomer feed composition

Table S1: impact of acrylic acid (AA) content in the monomer feed on degree of grafting (*DG*) and conductivity.

sample	AA content / mol–%	*DG* / %	*conductivity* / $mS \cdot cm^{-1}$
PVDF–AA–30	30	31	9.7
PVDF–AA–40	40	200	43.4
PVDF–AA–50	50	356	44.1
PVDF–AA–60	60	456	53.4
PVDF–AA–70	70	495	51.6
PVDF–AA–80	80	901	45.3
PVDF–AA–90	90	970	23.2
PVDF–AA–100	100	946	2.2

Variation of monomer fraction

Table S2: Impact of monomer volume fraction on *DG* and conductivity.

sample	*monomer fraction* / vol.–%	*DG* / %	*conductivity* / $mS \cdot cm^{-1}$
PVDF–M–10	10	28	0.3
PVDF–M–15	15	310	15.5
PVDF–M–20	20	356	44.1
PVDF–M–25	25	953	53.9
PVDF–M–30	30	1335	93.5

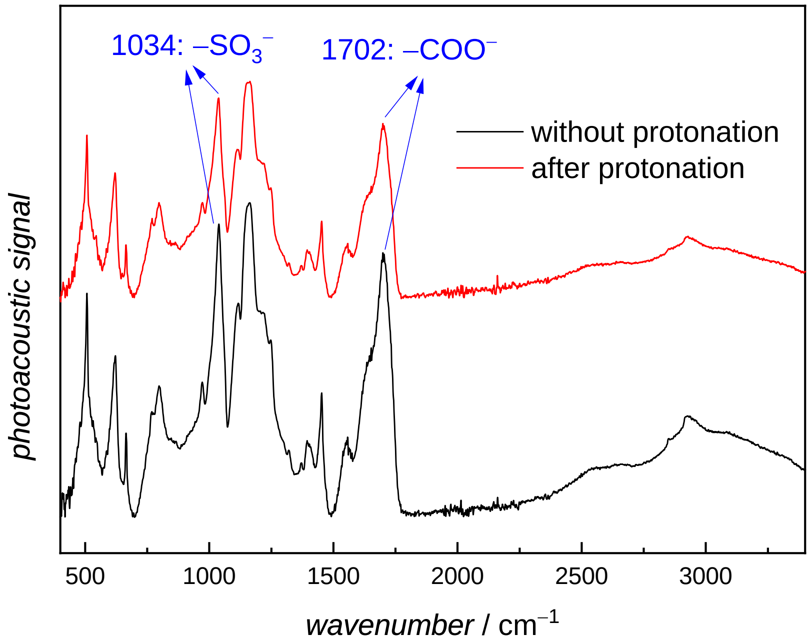

Figure S1: FTIR spectra of sample xx before and after protonation. The peaks assigned to $SO3^-$ and COO^- may be assigned to AMPS and AA units, respectively, in the graft copolymer.

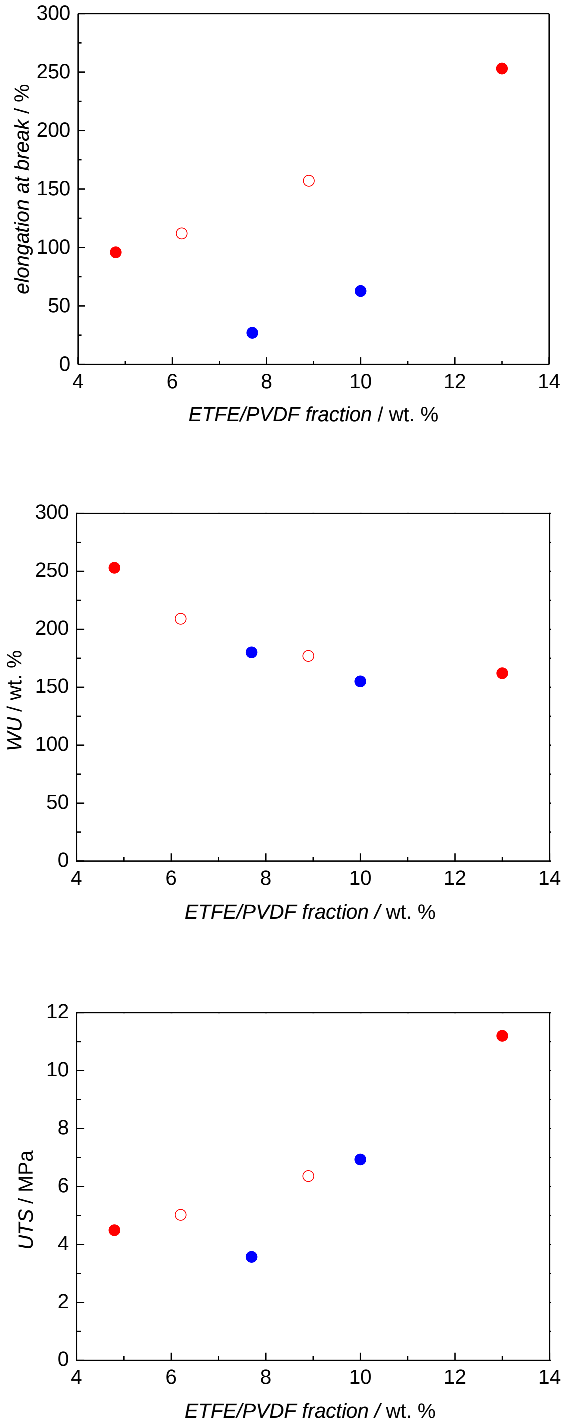

Figure S2: Variation of elongation at break, water uptake and ultimate tensile strength with the fraction of base material (ETFE or PVDF).

- blue points: GH220, GH170 (ETFE base material, GMA/HEMA comonomers)
- full red points: AAE205, AAE500 (ETFE base material, AMPS/AA comonomers)
- open red point: AAP305, AAP421 (PVDF base material, AMPS/AA comonomers)
